# The application value of 24 h Holter monitoring indices in predicting MACEs outside the hospital within three years after PCI in patients with STEMI

**DOI:** 10.3389/fcvm.2024.1401343

**Published:** 2024-07-23

**Authors:** Bingxin Chen, Li Men, Hongli Wang, Long Yang, Mingxi Li, Jingcheng Hu, Ping Fan

**Affiliations:** ^1^ Department of Heart Function, State Key Laboratory of Pathogenesis, Prevention and Treatment of High Incidence Diseases in Central Asia, First Affiliated Hospital of Xinjiang Medical University, Urumqi, China; ^2^ State Key Laboratory of Pathogenesis, Prevention and Treatment of High Incidence Diseases in Central Asia, Clinical Medical Research Institute, The First Affiliated Hospital of Xinjiang Medical University, Urumqi, China; ^3^ Department of Pediatric Cardiothoracic Surgery, First Affiliated Hospital of Xinjiang Medical University, Urumqi, China; ^4^ Department of Clinical Medicine, Xinjiang Medical University, Urumqi, China

**Keywords:** STEMI, MACEs, Holter, heart rate variability, deceleration capacity

## Abstract

**Background:**

Evaluating cardiovascular risk in patients experiencing acute ST-elevation myocardial infarction (STEMI) and undergoing percutaneous coronary intervention (PCI) is crucial for early intervention and improving long-term outcomes. 24 h Holter monitoring provides continuous cardiac electrophysiological data, enabling the detection of arrhythmias and autonomic dysfunction that are not captured during routine examinations. This study aimed to examine the relationship between Holter monitoring metrics and the occurrence of out-of-hospital major adverse cardiovascular events (MACEs) following PCI in patients with STEMI, offering insights into cardiovascular risk evaluation.

**Methods:**

This prospective cohort study included STEMI patients undergoing PCI. 24 h Holter monitoring data were recorded, including heart rate, heart rate variability (HRV) metrics such as SDNN and SDANN index, heart rate deceleration capacity (DC) at different time scales (DC2, DC4, DC8), and the frequency of premature ventricular contractions (PVCs). Independent correlations between these indices and MACEs, as well as cardiovascular deaths, were investigated using multifactorial logistic regression. Predictive capacities were assessed through receiver operating characteristic (ROC) curves.

**Results:**

A total of 172 participants were enrolled in this study. Over the 3-year follow-up period, MACEs were observed in 57 patients, including 20 cases of cardiac death. In logistic regression models adjusted for confounding variables, SDNN [OR: 0.980; 95% CI: (0.967, 0.994); *p* = 0.005] and SDANN index [OR: 0.982; 95% CI: (0.969, 0.996); *p* = 0.009] were negatively associated with the incidence of MACEs. Conversely, the slowest heart rate [OR: 1.075; 95% CI: (1.022, 1.131); *p* = 0.005] and frequent PVCs [OR: 2.685; 95% CI: (1.204, 5.987); *p* = 0.016] demonstrated a positive association with MACEs. Furthermore, SDNN [OR: 0.957; 95% CI: (0.933, 0.981); *p* = 0.001], DC [OR: 0. 702; 95% CI: (0.526, 0.938); *p* = 0.017]) and DC4 [OR: 0.020; 95% CI: (0.001, 0.664); *p* = 0.029] were negatively associated with cardiac death. The ROC analysis results indicated that SDNN was an effective predictor of both MACEs [AUC: 0.688 (95% CI: 0.601–0.776)] and cardiac death [AUC: 0.752 (95% CI: 0.625–0.879)].

**Conclusion:**

HRV, DC metrics, and frequent PVCs obtained by 24 h Holter monitoring were associated with the risk of MACEs in STEMI patients. These metrics can help clinicians identify at-risk patients early so that timely interventions.

## Background

Cardiovascular diseases remain one of the leading causes of mortality worldwide, with acute ST-segment elevation myocardial infarction (STEMI) being among the most severe manifestations of acute coronary syndromes (1, 2). The pathogenesis typically involves rupture or erosion of coronary artery atheromatous plaques, followed by thrombus formation, leading to a dramatic reduction or cessation of myocardial blood flow. In recent years, percutaneous coronary intervention (PCI) has emerged as the gold standard treatment for STEMI patients, significantly improving both acute and long-term clinical outcomes (3). However, despite undergoing PCI treatment, some patients still face the risk of major adverse cardiovascular events (MACEs) after discharge, including angina, myocardial reinfarction, unplanned rehospitalization for revascularization, heart failure, and cardiovascular death (4, 5). Therefore, early identification and risk stratification of these high-risk patients to implement targeted interventions are crucial for improving long-term prognosis.

The 24 h ambulatory electrocardiogram (Holter monitoring) serves as a non-invasive cardiac monitoring technology, capable of continuously recording a patient's cardiac electrophysiological activity throughout the day (6). This monitoring technique is particularly valuable for revealing intermittent arrhythmias and the cardiac activity of patients with atypical chest pain, as these conditions may not be easily captured during routine electrocardiographic examinations (7, 8). Multiple studies have investigated the application value of Holter monitoring in various cardiac diseases. For example, heart rate variability (HRV) is associated with mortality risk in patients with cardiovascular diseases (9). Moreover, HRV can stratify the risk of arrhythmias in myocardial infarction patients (10, 11). The width of the QRS complex and ST-segment elevation are linked to short-term and long-term cardiovascular mortality post-PCI in STEMI patients (12). The number of pathological Q waves is associated with left ventricular systolic dysfunction in STEMI patients (13). Elevated T-wave alternans can predict non-sustained ventricular tachycardia post-PCI in STEMI patients (14). Therefore, for STEMI patients, Holter monitoring not only assesses arrhythmias after myocardial reperfusion but also evaluates long-term risks and guides adjustments in subsequent treatment plans (15, 16).

Despite these findings, comprehensive studies integrating Holter monitoring metrics for long-term prognosis in STEMI patients post-PCI remain scarce. Given this background, this study aimed to explore the predictive value of specific ECG metrics from 24 h Holter monitoring for out-of-hospital major adverse cardiovascular events (MACEs) in STEMI patients up to three years post-PCI. This research will not only provide clinicians with additional insights to refine management strategies for STEMI patients but also uncover novel pathways for early intervention in cardiovascular disease, holding substantial clinical importance for the prevention and treatment of such conditions.

## Materials and methods

### Study design

This study was designed as a prospective cohort study to assess the value of 24 h Holter monitoring metrics in predicting the occurrence of out-of-hospital MACEs in STEMI patients within three years after undergoing PCI. We recruited STEMI patients diagnosed and treated with PCI for acute chest pain admitted to the emergency department of the First Affiliated Hospital of Xinjiang Medical University between January 2019 and December 2020. This study complied with the Declaration of Helsinki, and the study protocol was approved by the Human Ethics Committee of the First Affiliated Hospital of Xinjiang Medical University (approval number: K202309-12). Written informed consent was obtained from the included participating homozygotes.

### Study population

Patients were eligible for inclusion in this study if they satisfied the following criteria: (1) They arrived at the emergency department experiencing an acute episode of chest pain; (2) They were 18 years of age or older; (3) They fulfilled the diagnostic criteria for STEMI, which comprised: clinical signs indicative of acute myocardial ischemia, for instance, chest pain persisting for over 20 min; electrocardiograms (ECGs) indicating newly emerged ST-segment elevation with ST-segment elevation amounting to ≥2.5 mm in two consecutive leads in males for the anterior wall, ≥1.5 mm in females, and ≥1 mm in other leads, or a new left bundle branch block; and elevated blood biochemical markers (such as cardiac troponin) aligning with myocardial injury criteria. (4) Undergoing percutaneous coronary intervention (PCI) within 12 h from the onset of symptoms; (5) Agreeing to partake in the study and signing an informed consent form.

Participants were excluded if they had any of the following conditions: (1) Psychiatric or psychological disorders; (2) Severe hepatic or renal dysfunction; (3) Suffering from other serious cardiac diseases such as severe heart valve disease, hypertrophic cardiomyopathy, congenital heart disease, etc.; (4) A history of myocardial infarction or myocardial revascularization (PCI or coronary artery bypass graft surgery-CABG); (5) Inability or failure to undergo Holter monitoring postoperatively; (6) Refusal to sign a written informed consent.

### Clinical data collection

Age, gender, ethnicity, risk factors (e.g., high blood pressure, diabetes, smoking, alcohol use), and medication use were recorded, and measurements of height, weight, and blood pressure were taken. The collection of blood specimens for laboratory analysis was done exclusively by trained nursing staff. Upon admission, 8 ml of venous blood was drawn from each patient to assess triglycerides, high-density lipoprotein cholesterol, low-density lipoprotein cholesterol, and serum creatinine. Patients’ left ventricular ejection fraction was measured using a bedside ultrasound machine. Body mass index (BMI) was calculated as weight in kilograms divided by the square of height in meters. Regular smokers in the past 6 months were defined as current smokers, and those who had quit smoking for more than 6 months were defined as “ex-smokers”. Alcohol drinkers were defined as those who had consumed 100 grams of alcoholic beverages at least once a week in the past month, and those who had not consumed alcohol for more than one month were defined as “ever drinkers”. Diabetes mellitus was defined as any of the following: (1) self-reported history of diabetes mellitus or taking hypoglycemic drugs or insulin; (2) fasting blood glucose level ≥126 mg/dl measured by fasting venous blood in the early morning of the second day of admission; and (3) glycosylated hemoglobin (HbA1c) ≥6.5%. Hypertension was defined as any of the following: (1) self-reported history of hypertension or being on antihypertensive medication; (2) blood pressure exceeding 140/90 mmHg measured on 3 consecutive occasions after rest.

### 24 h Holter monitoring

Within 24–48 h after patients underwent PCI treatment, a Holter monitoring device (MedEx MECG-200, Beijing, China) was attached. Patients were equipped with a lightweight device fitted with multiple electrodes, which were connected to the device via wires and adhered to specific locations on the patient's chest. During the monitoring period, patients were requested to continue their daily activities to ensure that the data accurately reflected their cardiac status. After continuous monitoring for one day, the device was retrieved and the following metrics were recorded: maximum heart rate, minimum heart rate, average heart rate, and several parameters related to heart rate variability (HRV) including standard deviation of all NN intervals (SDNN), average of the standard deviations of NN intervals for each 5 min segment (SDNN index), standard deviation of the averages of NN intervals (SDANN index), number of pairs of adjacent NN intervals differing by more than 50 ms (NN50), root mean square of the successive differences between adjacent NN intervals (rMSSD), proportion of NN50 count to total NN intervals (pNN50), and the trigonometric index, which is the total number of NN intervals divided by the height of the NN interval histogram. Additionally, spectral analysis components such as total power (TP), ultra-low frequency (ULF), very low frequency (VLF), low frequency (LF), high frequency (HF), and low frequency to high frequency ratio (LF/HF ratio) were recorded. Additionally, the assessment of ventricular late potentials included metrics such as the width of the QRS complex, the duration of low amplitude signals below 40 microvolts following the end of the QRS complex, and the root mean square voltage within 40 milliseconds after the end of the QRS complex. Other significant ECG parameters such as the QT interval, mean RR interval, mean corrected QT interval (Mean QTc interval), QT interval dispersion (QTcD), deceleration capacity (DC), and deceleration capacity at different time scales (DC2, DC4, DC8) were also meticulously recorded. To further evaluate the arrhythmic risk, the frequency of premature ventricular contractions (PVCs) and the occurrence of non-sustained ventricular tachycardia (NSVT) were also analyzed. Frequent PVCs were defined as more than 30 PVCs per hour, and NSVT was defined as three or more consecutive ventricular beats at a rate of over 100 beats per min, lasting less than 30 s.

### Follow-up assessment

From the day of PCI treatment, patients were enrolled in a three-year follow-up program. We provided patients with contact information and conducted follow-ups through telephone calls and outpatient visits. The primary focus of the follow-up was the documentation of major adverse cardiovascular events (MACEs), including non-fatal myocardial infarction, heart failure, unplanned coronary revascularization, cardiogenic shock, malignant arrhythmias, gastrointestinal bleeding, and cardiovascular death. The occurrence of any of these events was considered a MACE, which terminated the follow-up. Multiple MACEs could occur in the same patient during the follow-up period.

### Statistical analysis

Initially, participants were categorized into two groups according to the occurrence of MACEs, and their baseline characteristics were compared. Normally distributed continuous variables were presented as mean ± standard deviation (SD), while non-normally distributed continuous variables were displayed as the median and interquartile range (25th and 75th percentiles). Comparisons between groups were conducted using the independent samples *t*-test or Mann–Whitney *U* test. Categorical variables were depicted as frequencies (percentages), with group comparisons performed using the chi-square test. Logistic regression analysis was utilized to investigate independent associations between 24 h Holter monitoring metrics and MACEs. The predictive capacity of various metrics for the occurrence of MACEs in STEMI patients was evaluated through receiver operating characteristic (ROC) curves. All the aforementioned analyses were conducted using R software (version 4.3.1), with a two-sided *P* value of less than 0.05 deemed statistically significant.

## Results

### Baseline characteristics of participants

**Figure 1:**
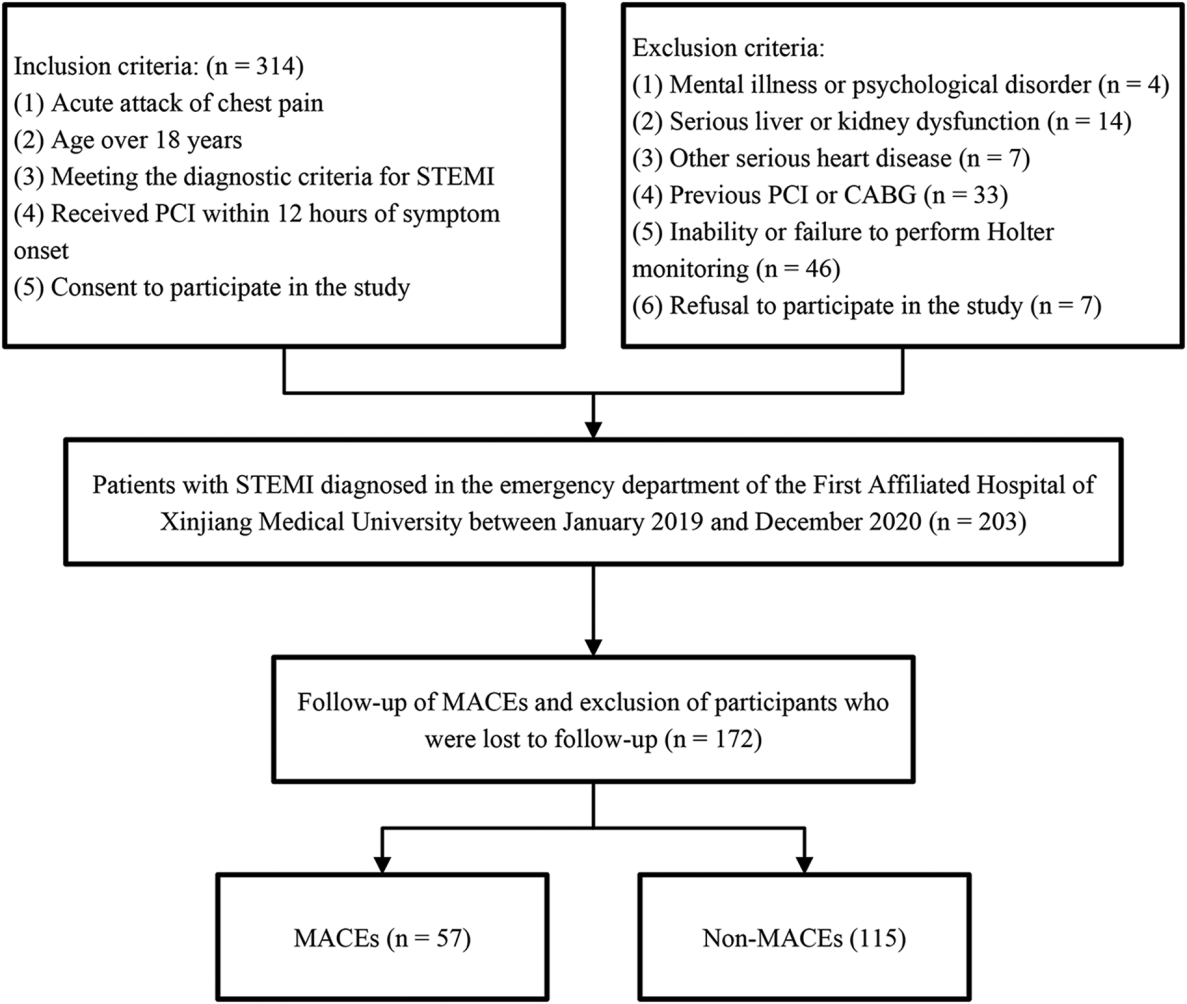
Flowchart for inclusion-exclusion of study design.

In this study, a total of 314 patients with STEMI meeting the inclusion criteria were initially screened. After the preliminary screening, 111 patients who did not meet the research requirements were excluded, leaving 203 patients enrolled in the follow-up study. During the follow-up process, 31 patients (accounting for 15.2%) were lost to follow-up, hence, the study was completed with 172 participants. During this period, 57 patients experienced MACEs, including 20 cases of cardiac death (Figure 1).

The study cohort included 26 females (15.1%) and 146 males (84.9%), with a median age of 59 years. Comparing the baseline characteristics of patients who did and did not experience MACEs, we found that those who experienced MACEs were older, had a higher prevalence of hypertension, and had lower levels of triglycerides and LVEF (*P* < 0.05). However, no significant statistical differences were observed between the two groups in terms of gender, ethnic background, smoking and drinking habits, diabetes prevalence, medication usage, BMI, blood pressure, HDL-C, LDL-C, and serum creatinine levels (*P* > 0.05). Further analysis of the 24 h Holter monitoring data revealed significant statistical differences between the two groups in parameters such as the lowest heart rate, SDNN, trigonometric index, SDANN index, LF, width of QRS, DC, DC2, DC4, DC8, and frequent PVCs (*P* < 0.05) (Table 1).

**Table 1:** Baseline characteristics of participants grouped by whether or not MACE occurred.

Characteristic	Total (*n* = 172)	Non-MACE (*n* = 115)	MACE (*n* = 57)	Statistic	*p*-value
Demographic information					
Age, years	59.0 (51.0, 68.0)	57.0 (49.0, 64.0)	67.0 (57.0, 74.0)	−4.131	<0.001
Sex					
Male	26 (15.1)	16 (13.9)	10 (17.5)	0.392	0.531
Female	146 (84.9)	99 (86.1)	47 (82.5)		
Ethnic					
Han	108 (62.8)	74 (64.3)	34 (59.6)	1.955	0.582
Kazakh	7 (4.1)	3 (2.6)	4 (7.0)		
Other	18 (10.5)	12 (10.4)	6 (10.5)		
Uighur	39 (22.7)	26 (22.6)	13 (22.8)		
Smoking					
Now	65 (37.8)	46 (40.0)	19 (33.3)	5.579	0.061
Never	83 (48.3)	58 (50.4)	25 (43.9)		
Former	24 (14.0)	11 (9.6)	13 (22.8)		
Drinking					
Now	44 (25.6)	31 (27.0)	13 (22.8)	1.804	0.406
Never	116 (67.4)	78 (67.8)	38 (66.7)		
Former	12 (7.0)	6 (5.2)	6 (10.5)		
Hypertension	92 (53.5)	53 (46.1)	39 (68.4)	7.641	0.006
Diabetes	33 (19.2)	19 (16.5)	14 (24.6)	1.589	0.208
Previous CHD	124 (72.1)	87 (75.7)	37 (64.9)	2.185	0.139
Previous heart attack	69 (40.1)	46 (40.0)	23 (40.4)	0.002	0.965
Drug utilization					
ACEI/ARB	75 (43.6)	55 (47.8)	20 (35.1)	2.515	0.113
Diuretic	14 (8.1)	9 (7.8)	5 (8.8)	0.046	0.831
Beta-blocker	91 (52.9)	65 (56.5)	26 (45.6)	1.82	0.177
CCB	29 (16.9)	19 (16.5)	10 (17.5)	0.028	0.866
Statin	107 (62.2)	74 (64.3)	33 (57.9)	0.675	0.411
Anti-hyperglycemic	13 (7.6)	6 (5.2)	7 (12.3)	2.721	0.099
Insulin	10 (5.8)	4 (3.5)	6 (10.5)	3.457	0.063
Clinical indicators					
BMI, kg/m^2^	26.1 ± 3.5	26.5 ± 3.3	25.5 ± 3.6	1.805	0.073
SBP, mmHg	122.0 (111.0, 131.0)	120.0 (108.0, 130.0)	127.0 (114.0, 137.0)	−1.654	0.098
DBP, mmHg	75.0 (66.0, 81.0)	75.0 (67.0, 81.0)	75.0 (66.0, 83.0)	−0.14	0.89
Triglyceride, mmol/L	1.35 (1.00, 2.09)	1.41 (1.00, 2.37)	1.21 (0.94, 1.72)	2.012	0.044
HDL-C, mmol/l	0.90 (0.76, 1.08)	0.91 (0.77, 1.09)	0.89 (0.73,1.07)	1.272	0.204
LDL-C, mmol/l	1.90 (1.57,2.37)	1.89 (1.57,2.35)	1.91 (1.54,2.47)	−0.465	0.643
Creatinine, mmol/l	75.5 (66.0, 88.0)	75.0 (66.9, 85.4)	77.0 (61.0, 90.6)	−0.42	0.676
LVEF, %	59.8 (53.4, 62.4)	60.0 (56.0, 63.0)	57.0 (50.5, 60.9)	3.084	0.002
Ambulatory electrocardiogram information					
Fastest heart rate	105.0 (95.0, 116.0)	106.0 (95.0, 116.0)	104.0 (94.0, 116.0)	0.213	0.832
Slowest heart rate	52.0 (47.0, 58.0)	51.0 (45.0, 56.0)	57.0 (50.0, 60.0)	−3.567	<0.001
Average heart rate	71.0 (64.0, 77.0)	70.0 (64.0, 76.0)	72.0 (66.0, 79.0)	−1.402	0.161
Heart rate variability					
SDNN	100.8 (78.3, 119.4)	105.1 (86.2, 127.2)	79.1 (61.5, 107.9)	4.016	<0.001
Trigonometric index	34.6 (22.6, 53.6)	41.4 (23.2, 62.1)	28.2 (21.4, 37.9)	3.479	<0.001
SDNN index	23.8 (16.8, 32.8)	23.8 (18.4, 33.6)	22.6 (15.7, 31.5)	0.67	0.504
SDANN index	85.8 (63.0, 107.8)	90.8 (71.2, 112.2)	69.1 (49.6, 98.0)	3.573	<0.001
NN50	4,058.0 (1,365.0, 8,113.0)	4,167.0 (1,479.0, 8,700.0)	3,548.0 (1,287.0, 6,699.0)	0.916	0.361
rMSSD	27.8 (21.0, 37.9)	28.2 (22.1, 37.4)	27.7 (20.8, 38.2)	0.216	0.83
pNN50	4.220 (1.580, 9.720)	5.090 (1.540, 11.370)	3.610 (1.580, 8.860)	0.992	0.322
TP	2,156.4 (1,114.5, 5,469.2)	2,287.5 (1,132.4, 6,117.3)	1,947.7 (844.3, 5,280.8)	0.803	0.423
ULF	803.7 (153.4, 3,200.8)	902.1 (178.3, 3,371.6)	654.9 (91.8, 3,041.0)	0.849	0.397
VLF	1,207.4 (422.7, 2,626.5)	1,290.6 (526.2, 2,694.3)	983.3 (306.1, 2,583.1)	1.104	0.27
LF	166.9 (77.5, 328.9)	203.5 (88.6, 386.0)	108.5 (53.0, 227.2)	3.141	0.002
HF	68.8 (35.5, 156.8)	71.4 (40.1, 175.3)	58.4 (33.4, 148.7)	1.235	0.218
LF: HF	2.290 (1.270, 3.990)	2.540 (1.360, 4.210)	1.980 (1.020, 3.450)	1.846	0.065
Ventricular late potential					
Width of QRS	89.0 (80.0, 103.0)	87.0 (77.0, 98.0)	98.0 (84.0, 117.0)	−2.765	0.006
Intervals below 40 µv	34.0 (26.0, 44.0)	34.0 (25.0, 43.0)	37.0 (27.0, 46.0)	−1.257	0.209
40 ms rms voltage after QRS	140.3 ± 58.7	136.8 ± 57.3	147.5 ± 60.9	−1.122	0.263
QT					
Mean RR	759.0 (638.0, 895.0)	759.0 (618.0, 923.0)	731.0 (638.0, 882.0)	−0.176	0.862
Mean QTc interval	388.0 (308.0, 422.0)	388.0 (325.0, 417.0)	383.0 (255.0, 427.0)	−0.018	0.987
QTcD	892.0 (701.0, 1,012.0)	866.0 (610.0, 1,012.0)	906.0 (770.0, 1,014.0)	−1.015	0.311
Deceleration capacity					
DC	5.516 ± 2.386	5.806 ± 2.481	4.930 ± 2.061	2.286	0.023
DC2	7.298 ± 2.178	7.573 ± 2.240	6.742 ± 1.933	2.38	0.018
DC4	0.350 (0.230, 0.540)	0.390 (0.260, 0.620)	0.260 (0.130, 0.410)	3.11	0.002
DC8	0.010 (0.000, 0.030)	0.020 (0.000, 0.040)	0.010 (0.000, 0.020)	2.885	0.003
Holter outcome assessment					
Late ventricular potentials (positive)	16(9.3)	10(8.7)	6(10.5)	0.151	0.697
Frequent PVC	54(31.4)	25(21.7)	29(50.9)	15.023	<0.001
NSVT	42(24.4)	30(26.1)	12(21.1)	0.523	0.469

BMI, body mass index; SBP, systolic blood pressure; DBP, diastolic blood pressure; HDL-C, high-density lipoprotein cholesterol; LDL-C, low-density lipoprotein cholesterol; LVEF, left ventricular ejection fraction; SDNN, standard deviation of NN intervals; SDNN index, average of the standard deviations of NN intervals for each 5 min segment; SDANN index, standard deviation of the averages of NN intervals; NN50, number of pairs of adjacent NN intervals differing by more than 50 ms; rMSSD, root mean square of the successive differences between adjacent NN intervals; pNN50, proportion of NN50 count to total NN intervals; TP, total power; ULF, ultra-low frequency; VLF, very low frequency; LF, low frequency; HF, high frequency; LF:HF, low frequency to high frequency ratio; QRS, QRS complex width; QTc, corrected QT interval; QTcD, QT interval dispersion; DC, deceleration capacity; DC2, DC4, DC8, deceleration capacity at different time scales; PVC, premature ventricular contractions; NSVT, non-sustained ventricular tachycardia.

### Association of 24 h Holter monitoring indicators with STEMI occurrence of MACEs

In this study, we undertook logistic regression analyses on the 24 h Holter monitoring indicators that demonstrated statistical differences in the baseline data. The initial univariate logistic regression analysis revealed that indicators such as SDNN, Trigonometric index, SDANN index, DC, DC2, DC4, and DC8 exhibited a negative correlation with the incidence of MACEs, indicated by OR less than 1. Conversely, the Slowest Heart Rate, Width of QRS, and frequent premature ventricular beats showed a positive correlation with MACE occurrence, denoted by OR greater than 1. To delve deeper into the independent associations of these indicators with MACEs while considering potential confounders, we performed multivariate logistic regression analyses, adjusting for established cardiovascular risk factors. The findings from this analysis highlighted that SDNN [OR: 0.980; 95% CI: (0.967, 0.994); *p* = 0.005] and SDANN index [OR: 0.982; 95% CI: (0.969, 0.996); *p* = 0.009] maintained their negative association with the occurrence of MACEs. In contrast, the Slowest Heart Rate [OR: 1.075; 95% CI: (1.022, 1.131); *p* = 0.005] and frequent PVCs [OR: 2.685; 95% CI: (1.204, 5.987); *p* = 0.016] were positively associated with MACEs (Table 2). Furthermore, our investigation extended to analyzing the relationship between 24-hour Holter monitoring indicators and cardiac death. In the logistic regression model adjusted for covariates, the Slowest Heart Rate emerged as a positive predictor of cardiac death [OR: 1.084; 95% CI: (1.007, 1.166); *p* = 0.031]. Conversely, SDNN [OR: 0.957; 95% CI: (0.933, 0.981); *p* = 0.001], SDANN index [OR: 0.966; 95% CI: (0.944, 0.989); *p* = 0.004], DC [OR: 0.702; 95% CI: (0.526, 0.938); *p* = 0.017], and DC4 [OR: 0.020; 95% CI: (0.001, 0.664); *p* = 0.029] were inversely associated with cardiac death (Table 3).

**Table 2:** Logistic regression of factors influencing the occurrence of MACE in STEMI patients.

Characteristic	OR (95% CI)	*P*-value	OR (95% CI)-adjusted	*P*-value-adjusted
Slowest heart rate	1.086 [1.038, 1.136]	<0.001	1.075 [1.022, 1.131]	0.005
SDNN	0.978 [0.966, 0.990]	<0.001	0.980 [0.967, 0.994]	0.005
Trigonometric index	0.970 [0.953, 0.987]	0.001	1.001 [0.999, 1.002]	0.228
SDANN index	0.981 [0.970, 0.992]	0.001	0.982[0.969,0.996]	0.009
LF	0.998 [0.997, 1.000]	0.059	0.999 [0.998, 1.001]	0.270
Width of QRS	1.018 [1.004, 1.032]	0.013	1.012 [0.996, 1.028]	0.139
DC	0.856 [0.746, 0.981]	0.026	0.886 [0.745, 1.054]	0.172
DC2	0.141 [0.034, 0.584]	0.007	0.940 [0.783, 1.128]	0.506
DC4	0.837 [0.720, 0.973]	0.021	0.814 [0.153, 4.339]	0.810
DC8	0.546 [0.351, 0.801]	0.004	0.654 [0.413, 1.035]	0.070
Frequent PVC	3.729 [1.884, 7.379]	<0.001	2.685 [1.204, 5.987]	0.016

“adjusted” indicates that each variable was adjusted for age, BMI, Hypertension, Previous CHD, SBP, Triglyceride, LVEF, ACEI/ARB, Beta-blocker, and Anti-hyperglycemic.

OR, odds ratio; CI, confidence interval; SDNN, standard deviation of NN intervals; SDNN index, average of the standard deviations of NN intervals for each 5 min segment; SDANN index, standard deviation of the averages of NN intervals; LF, low frequency; QRS, QRS complex width; DC, deceleration capacity; DC2, DC4, DC8, deceleration capacity at different time scales; PVC, premature ventricular contractions.

**Table 3:** Logistic regression of factors influencing the occurrence of death in STEMI patients.

Characteristic	OR (95% CI)	*P*-value	OR (95% CI)-adjusted	*P*-value-adjusted
Slowest heart rate	1.080 [1.017, 1.147]	0.013	1.084 [1.007, 1.166]	0.031
SDNN	0.964 [0.945, 0.983]	<0.001	0.957 [0.933, 0.981]	0.001
Trigonometric index	1.000 [0.998, 1.002]	0.849	0.999 [0.997, 1.002]	0.614
SDANN index	0.973 [0.955, 0.991]	0.003	0.966 [0.944, 0.989]	0.004
LF	0.999 [0.997, 1.001]	0.388	1.000 [0.998, 1.002]	0.776
Width of QRS	1.011 [0.994, 1.028]	0.220	1.003 [0.982, 1.025]	0.776
DC	0.770 [0.632, 0.938]	0.009	0.702 [0.526, 0.938]	0.017
DC2	0.835 [0.677, 1.031]	0.094	0.898 [0.688, 1.172]	0.427
DC4	0.009 [0.000, 0.175]	0.002	0.020 [0.001, 0.664]	0.029
DC8	0.367 [0.158, 0.849]	0.019	0.457 [0.184, 1.137]	0.092
Frequent PVC	3.929 [1.500, 10.287]	0.005	2.295 [0.744, 7.080]	0.148

“adjusted” indicates that each variable was adjusted for age, BMI, Hypertension, Previous CHD, SBP, Triglyceride, LVEF, ACEI/ARB, Beta-blocker, and Anti-hyperglycemic.

OR, odds ratio; CI, confidence interval; SDNN, standard deviation of NN intervals; SDNN index, average of the standard deviations of NN intervals for each 5 min segment; SDANN index, standard deviation of the averages of NN intervals; LF, low frequency; QRS, QRS complex width; DC, deceleration capacity; DC2, DC4, DC8, deceleration capacity at different time scales; PVC, premature ventricular contractions.

### Predictive value of 24 h Holter monitoring for MACEs in STEMI patients

**Figure 2:**
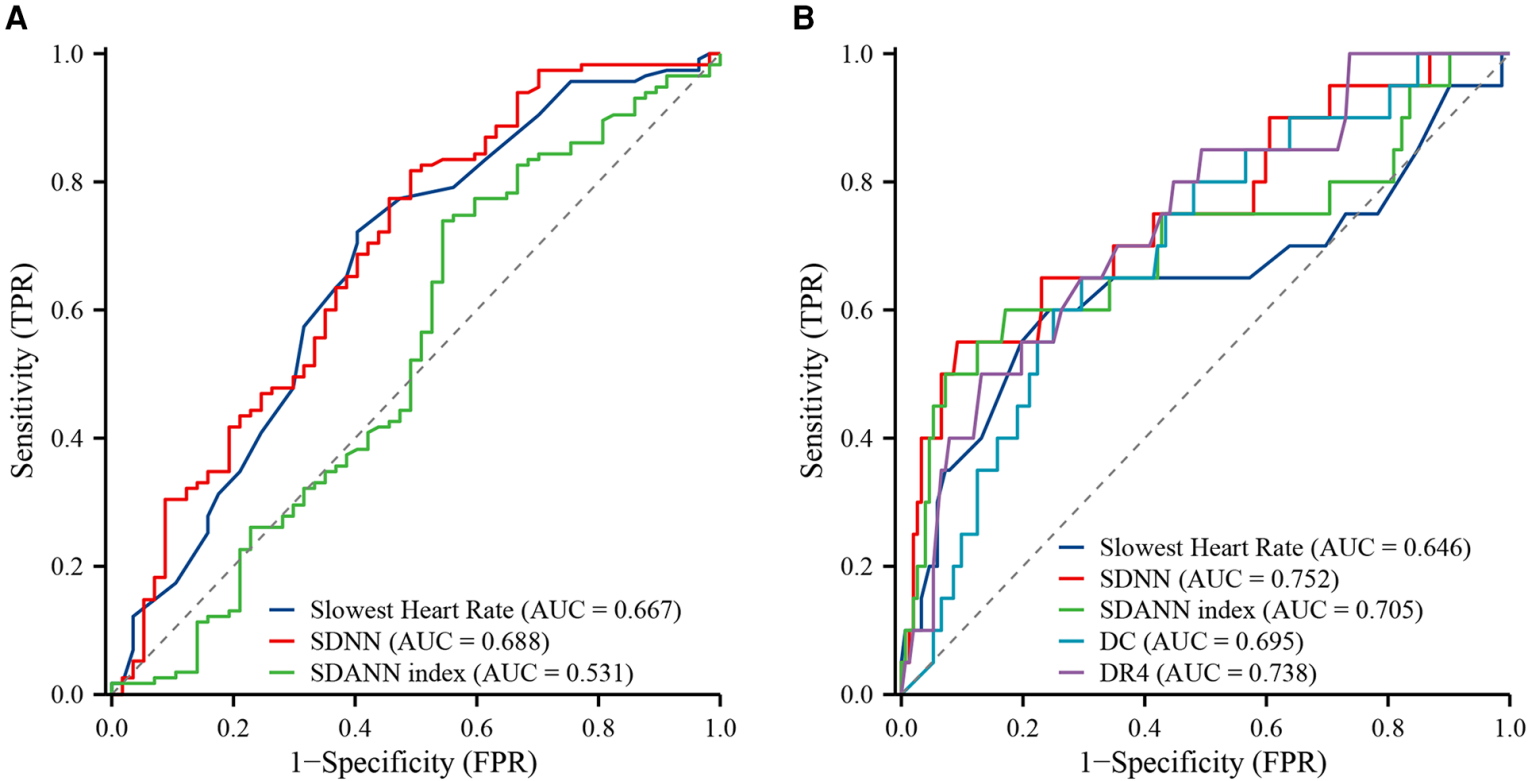
ROC curves of 24 h Holter monitoring metrics for the occurrence of MACEs and cardiovascular death in STEMI patients. (**A**) MACEs; (**B**) cardiac death.

The ROC analysis was conducted to evaluate the predictive value of selected 24-hour Holter monitoring indicators for MACEs in STEMI patients (Table 4). The analysis revealed that the Slowest Heart Rate demonstrated a moderate discriminative ability with an AUC of 0.667 (95% CI: 0.578–0.757), achieving a sensitivity of 72.2% and specificity of 59.6% at the optimal cut-off value of 55.5 beats per min. The SDNN indicator showed a slightly higher predictive performance, with an AUC of 0.688 (95% CI: 0.601–0.776), and was able to identify MACEs with a sensitivity of 81.7% and specificity of 50.9% at a cut-off value of 79.7 ms. In contrast, the SDANN index exhibited limited utility in predicting MACEs, with an AUC of 0.531 (95% CI: 0.433–0.630), a sensitivity of 73.9%, and a specificity of 45.6% at the cut-off value of 19.315 ms. These findings indicate that the Slowest Heart Rate and SDNN are relatively better predictors for MACE occurrence in this patient population, the SDANN index demonstrates marginal predictive value (Figure 2A). Notably, SDNN was a strong predictor of cardiac death with an AUC of 0.752 (95% CI: 0.625–0.879), a sensitivity of 55.0%, and a high specificity of 90.8% at the cut-off of 65.875 ms. Additionally, DC4 stood out with an AUC of 0.738 (95% CI: 0.621–0.854), a sensitivity of 50.0%, and a notably high specificity of 86.8% at a 0.145 cut-off (Figure 2B).

**Table 4:** ROC analysis of ambulatory electrocardiographic indices to predict postoperative occurrence of MACE and death in patients with STEMI.

Characteristic	AUC	95% CI	Sensitivity	Specificity	Cut-off value
MACE					
Slowest heart rate	0.667	0.578–0.757	0.722	0.596	55.500
SDNN	0.688	0.601–0.776	0.817	0.509	79.700
SDANN index	0.531	0.433–0.630	0.739	0.456	19.315
Death					
Slowest heart rate	0.646	0.485–0.807	0.600	0.757	57.500
SDNN	0.752	0.625–0.879	0.550	0.908	65.875
SDANN index	0.705	0.555–0.855	0.600	0.829	60.900
DC	0.695	0.577–0.814	0.650	0.704	4.605
DC4	0.738	0.621–0.854	0.500	0.868	0.145

AUC, area under the curve; CI, confidence interval; SDNN, standard deviation of NN intervals; SDNN index, average of the standard deviations of NN intervals for each 5 min segment; SDANN index, standard deviation of the averages of NN intervals; DC, deceleration capacity; DC4, deceleration capacity at 4 s time scale.

## Discussion

In this study, we identified significant correlations between several key metrics from 24 h Holter monitoring and the occurrence of major adverse cardiovascular events (MACEs) and cardiac death in STEMI patients post-PCI. Specifically, reduced SDNN and SDANN index were inversely related to an elevated risk of MACEs, while the higher slowest heart rate and frequent PVCs were positively correlated with MACEs. For cardiac death, SDNN, SDANN index, DC, and DC4 were protective, whereas a higher slowest heart rate indicated increased risk.

STEMI arises from acute and sustained coronary artery hypoxia and ischemia, leading to corresponding myocardial necrosis (17). Typically, cardiovascular disease patients exhibit varying degrees of increased heart rate after onset, which serves as a crucial diagnostic marker post-PCI for STEMI patients, aiding in the assessment of patient recovery (18, 19). Our study's results indicated that an elevated lowest heart rate was associated with the occurrence of MACEs. This finding was consistent with previous studies, suggesting that a higher minimum heart rate may reflect reduced parasympathetic (vagal) tone and/or increased sympathetic activity (20–22). This autonomic dysregulation can lead to increased myocardial oxygen demand, reduced myocardial perfusion, and heightened electrical instability, ultimately resulting in MACEs. Clinically, STEMI and arrhythmias can exacerbate each other, compounding disease progression and increasing clinical mortality rates (23). This study also demonstrated that frequent premature ventricular contractions (PVCs) are positively correlated with the occurrence of MACEs in STEMI patients post-PCI. PVCs, which were early depolarizations originating from the ventricles, were a common form of arrhythmia and could disrupt the normal sequence of cardiac contraction, leading to reduced cardiac efficiency and increased myocardial oxygen demand (24). Frequent PVCs indicated underlying myocardial instability and electrical heterogeneity, both of which were markers of increased arrhythmic risk (10, 25, 26).

HRV reflects the variation in intervals between heartbeats, incorporating information about neurohumoral factors’ regulation of the cardiovascular system. Hence, HRV may serve as a valuable indicator for predicting sudden cardiac death and arrhythmic events (27). Reduced HRV indicates impaired autonomic nervous function, a common condition among patients with certain structural heart diseases such as congestive heart failure and acute myocardial infarction (28). Our study showed that SDNN and the SDANN index were associated with MACEs and cardiovascular mortality, and SDNN demonstrated moderate predictive capability. SDNN reflects the overall influence of HRV, including autonomic regulation of cardiac rhythm and/or rate, and intuitively indicates the extent of HRV (29). SDANN index estimates the long-term components of heart rate variability (30). In addition to autonomic activity, diurnal rhythms such as body temperature and the renin-angiotensin system also contribute to this variability measure (31). A decline in SDNN suggests an imbalance in the autonomic nervous system, possibly due to excessive activation of the sympathetic nervous system and reduced parasympathetic activity, thereby increasing the risk of arrhythmias and other cardiovascular events (32, 33). Furthermore, a reduction in SDNN may reflect instability in the cardiac repolarization process, elevating the likelihood of adverse cardiovascular events (34). Persistent imbalance in the autonomic nervous system not only poses an immediate health threat but may also drive the progression of cardiovascular diseases, leading to ongoing structural and functional damage (35). Therefore, SDNN serves not only as an indicator of short-term risk for STEMI patients but may also signify potential long-term cardiovascular health risks. Previous studies have shown that time-domain parameters such as SDNN and SDANN index, as well as frequency-domain parameters like LF and HF, are associated with the prognosis of STEMI patients (36, 37). However, our study did not observe significant differences in frequency-domain parameters between the MACEs and non-MACEs groups. The reason may be that frequency-domain metrics are more sensitive to short-term changes in autonomic balance and may not effectively capture long-term autonomic regulation as time-domain metrics do. Additionally, frequency-domain parameters are susceptible to various confounding factors such as respiration, physical activity, and measurement conditions, which might reduce their reliability in predicting long-term outcomes (38, 39).

In this study, DC had a moderate predictive value for cardiovascular death. A notable advantage of DC is its measurement impartiality to external factors and premature beats (40). Utilizing phase rectified signal averaging techniques to extract and detect variations in each cardiac cycle and its regulatory traces offers an objective reflection of the autonomic nervous system's direct regulatory effect on heart rate, including a quantitative analysis of vagal activity (41). The study revealed that the AUC for DC was 0.695, while for DR4, it was 0.738. DC primarily measures the capacity for heart rate reduction, reflecting the heart's response to parasympathetic nervous activation (42). Within the context of cardiovascular health, the parasympathetic nervous system plays a protective role by lowering heart rate and reducing cardiac metabolic demand, thus alleviating cardiac burden (43). A high DC value typically indicates robust parasympathetic regulatory capability, suggesting the heart's effective stress response and reduced risk of cardiovascular events (44). Conversely, a low DC value might indicate diminished parasympathetic function, associated with an increased risk of cardiovascular mortality. DC4, derived from the analysis of dynamic heart rate changes, is typically employed to assess the speed and extent of heart rate recovery (45). Rapid heart rate recovery signifies healthy autonomic nervous system function and good cardiovascular adaptability, whereas slow recovery may indicate an imbalance in the autonomic nervous system, especially due to excessive sympathetic activity and insufficient parasympathetic function (46, 47). Therefore, as an indicator of heart rate recovery capability, a decrease in DR4 is associated with increased cardiovascular mortality, reflecting a diminished cardiac stress response and overall reduced cardiovascular system adaptability.

### Advantages and limitations

Currently, there is a lack of research on the significance of 24 h Holter monitoring indicators for the specific cohort of patients admitted with acute chest pain and subsequently diagnosed with STEMI undergoing PCI. This study, through a comprehensive analysis of 24 h Holter monitoring data, delves into the correlation between various dynamic ECG indicators and cardiovascular events, providing new insights into cardiovascular risk assessment. This method facilitates a detailed evaluation of patient autonomic nervous system function, thereby enhancing the predictive value of the research. While this study offers valuable insights, the relatively small sample size may limit the generalizability of the results. Future research is needed in a larger patient population to validate these findings and strengthen the reliability and representativeness of the conclusions. As an observational study, potential confounding factors that could not be eliminated might affect the interpretation of the results. Despite efforts to adjust for these factors through multivariate analysis, it remains challenging to eliminate all potential biases. During follow-up, the inability to convert relative risk to absolute risk as well as the inability to differentiate between sudden and non-sudden cardiovascular deaths due to the inability of the majority of patients to recall the exact time of the event and the specifics of the event suggests the need for more detailed follow-up in future studies to assess the prognosis of STEMI with more precise risk values.

## Conclusion

This finding highlighted the significant value of SDNN, SDANN index, slowest heart rate, DC, DC4, and frequent PVCs in predicting the occurrence of MACE and cardiac death in STEMI patients within three years after PCI. These metrics emphasize the importance of considering ECG ambulatory monitoring indices in the risk assessment and management of STEMI patients.

## Data Availability

The raw data supporting the conclusions of this article will be made available by the authors, without undue reservation.
